# Supplementary material for: HARLEY mitigates user bias and facilitates efficient quantification and co-localization analyses of foci in yeast fluorescence images
Source: Sci Rep. 2022 Jul 18;12:12238. doi: 10.1038/s41598-022-16381-2 (PMC9293886; doi:10.1038/s41598-022-16381-2)
Supplement: Supplementary file 7 — Supplementary Information 1. [file 41598_2022_16381_MOESM7_ESM.docx]

Supplementary video figure legends

Video 1:

Explanation of cell segmentation using HARLEY, corresponding to figure 2a. Various employed parameters and output formats are explained and a brief overview of HARLEYs algorithm is given.

Video 2:

Explanation of the pre-processing step using HARLEY, corresponding to figure 2b. This step is comprised of stacking fluorescence images, denoising them and finally aggregating the result into one aggregate *.cells file.

Video 3:

Explanation on how to train the SVM HARLEY uses to classify foci, corresponding to the right side of figure 2c. This video also elucidates how to practically assess model performance and the tools HARLEY offers for this.

Video 4:

Explanation of how to use the trained SVM to automatically quantify (and manually correct) a dataset of cells as well as explanations of the output files HARLEY offers. This corresponds to the left side of Figure 2c.

Video 5:

Explanation of how to use multiple previously classified datasets to perform a co-localization analysis using HARLEY. This video elaborates on the different graphs available to view the data as well as the final output tables and corresponds to figure 2d.

Video 6:

As an alternative to training an SVM, HARLEY can use simple thresholds on brightness to quantify foci. This corresponds to the dashed arrow in figure 2c and is applicable to datasets with very low levels of background signal.
