# Supplementary material for: HARLEY mitigates user bias and facilitates efficient quantification and co-localization analyses of foci in yeast fluorescence images
Source: Sci Rep. 2022 Jul 18;12:12238. doi: 10.1038/s41598-022-16381-2 (PMC9293886; doi:10.1038/s41598-022-16381-2)
Supplement: Supplementary file 8 — Supplementary Information 2. [file 41598_2022_16381_MOESM8_ESM.pdf]

HARLEY mitigates user bias and faciliates efficient quantification and co-localization analyses of foci in yeast fluorescence images

Ilya Shabaov and J. Ross Buchan

Supplementary Methods

Harley Parameter Overview

ver. 1.2.4

June, 8th 2022

- Harley Parameter Overview
  - Cell Detection Pipeline
    - Threshold
    - Cleaning
    - Cell Centers
    - Cell Fitting
  - Preprocessing
    - Stacking
    - Denoise
    - Cell Selection
  - Model Training
    - Foci Candidates
    - Labeling
    - Training
  - Foci Detection (via model)
    - Foci Detection
  - Foci Detection (via Parameters)
    - Foci Candidates
    - Foci Detection Params
  - Colocalization
    - Dataset Alignment
    - Cell Overview
    - Graphs
  - References

Cell Detection Pipeline

This pipeline detects yeast cells in an image and outputs a "*.mask" file that contains the geometrical coordinates of each cell. The Mask file is then used together with fluorescence data in the next step.

Threshold

Type

The choice of source image for thresholding.
"Raw Image": Use the grayscale image itself

"Smoothed Gradient": Use gradient image smoothened by a gaussian before threshold is applied.

"Ridges (recommended)": Will run Frangi ridge detection. The smoothing factor governs the approximate thickness of the edges.

Smoothing (only if Type is "Ridges" or "Smoothed Gradient")

Smoothing for Ridges refers to the Frangi smoothing parameter described in [1].

"Smoothened Gradient" refers to the strength of the Gaussian blur applied to the gradient image. Gradient Images will usually produce double lines around cell outlines, and smoothing can help to remove this. Set the smoothing factor high enough so that you do not see double outlines anymore, but rather fat outlines. The units are standard deviation in pixels for the gaussian smoothing kernel.

Threshold Range

Intensity is a range/spectrum between 0 and 1, 0 being black and 1 being white. Thresholding allows a user to pick out a subrange inside 0-1.

"Use Band" (recommended): Will discard everything outside of a subrange/band of the spectrum. For example discard all whites or all blacks.

"Discard Band": Will discard a subrange/band of the spectrum. This allows users to exclude all grays and keep strong whites and strong blacks.

Color of Cell Wall (only if type is "Ridges")

In most cases yeast cell walls captured in a polarized light image will be white, though in some cases, the outlines may appear black. Change this setting if your cell outlines are black.

Threshold (Looks slightly different if Threshold Range is Use or Discard Band):

Any image used here, be it the raw or processed image is essentially a grayscale image with values 0-1. This setting is the final threshold applied to the image.

In Raw Image: 0 being black and 1 being white
In Gradient Image: 0 being no change and 1 being strong change
In Ridge Image: 0 being no ridge and 1 being strong ridge probability

Note that the values usually need to be adjusted when you switch Type (Raw,Gradient,Ridge).

Cleaning

Small Blob Removal:

In order to make the thresholded image clearer, small blobs can be removed by limiting different values (see below). This step done correctly will increase the number of detected cells and the quality of cell detection.
The goal is to remove artifacts inside cells (e.g., vacuoles) and outside cells (e.g., debris) that are not the cell wall/membrane.

Eccentricity Range:

An eccentricity of 0 means the outer shape (convex hull) of a blob is circular. The higher it is the less circular (and more elongated) the blob is.

Size Range:

The min and max width/height of the bounding box of the blob.

Area Range:

The min and max number of white pixels in the blob.

Solidity Range:

The number of white pixels in the blob divided by the area of its convex hull. A convex blob will have a ratio of 1. Blobs with big holes (i.e., outlines) will get very small values.

Cell Centers

Cell Size

The min and max dimension of a cell in pixels. Try to keep this parameter tight to increase performance. Cells are detected by fitting an ellipse to the outlines detected via thresholding. These ellipses are limited in size by this parameter. If you don't detect big or very small cells, increase the cell size range.

Fast Mode

Fast mode works best for images that have clearer outlines. In order to determine cell centers, it first computes the distance of each pixel to the detected cell walls. This distance has to be between the minimum and maximum radius of cells, defined in the parameter above. Additionally, the algorithim scans around the peaks of this distance map. The downside is that in noisy images, you might miss a few cell centers. In Fast Mode, every stride is set automatically to 10 pixels.

In the majority of cases this is the recommended setting. Unless experiencing significant difficulties in identifying a high percentage of cell centers, don't disable this setting.

Min Boundary Percentage

The percentage of boundary that the algorithm needs in order to define valid cell candidates. A value of 0.8 means that the gaps in a supposed cell boundary are less than 20%. Increasing this parameter will increase quality and confidence of detected cells, but might increase false negatives (i.e., when the full cell outline was not detected) as well as make the algorithm slightly slower. Decreasing this parameter might also lead to empty spaces between several adjacent cells being recognized as cells.

Try out what works in your case, you always have the ability to manually reject detected cell candidates later.

Stride (only if Fast Mode is disabled)

To speed up the algorithm we can skip pixels in heatmap generation and approximate the heatmap in the skipped positions. A stride of 1 means no skipping. 2 means we skip every second pixel, which means the algorithm runs stride^2 times faster. It makes sense to first work with a big stride, find good parameters and then reduce it to a smaller value. However in practice even a big stride value will give you very good results. It is recommended that you use the “fast mode” setting, in which case this parameter is automatically set to 10 pixels.

Cell Fitting

Min Confidence

The heatmap (previous pipeline step) gives a confidence of 0-1, with 1 being the best. To reduce the influence of noise, you can set this parameter tighter to filter out false-positives. If the previous step is done correctly, the confidence is almost always ~1 at the peaks (i.e., cell centers) of cells and the algorithm therefore is generally insensitive to this parameter.

Min Cell Distance

Governs how close cells can be to one another. This depends solely on resolution. Within min cell distance there can only be one detected cell; that with the highest score will be chosen. In yeast images, cells rarely overlap and setting this parameter to a high value will simply ensure that only cells that are detected with more confidence make it into the final selection. Practically in most cases this parameter can be ignored and the selection of cells done later manually with a few clicks.

Min Peak Size

A size constraint for finding maxima in the heatmap (i.e., cell centers). A maxima needs to be at least this size to be considered one. Making this large will exclude small and less probable cell centers. However the cell distance constraint will in most cases eliminate most small maxima.Practically, in most cases with good source images, cell centers will be very clear and this parameter will have little effect. Use in only noisy images, or manually select the cells.

Boundary Snapping

Originally cells are estimated as (rotated and scaled) ellipses. In the last step the ellipses are snapped to the respective pixel outlines (from the thresholded image), to refine the cell shape. The snapping parameter governs how much snapping is happening. 0 means we retain ellipses, 1 represents a full snap to boundaries.

It is highly recommended to keep this parameter at 1, to capture the cell outline. However to understand how the algorithm fits ellipses, you can set it to 0 and see the intermediary step of “detecting ellipses”.

Preprocessing

Preprocesses the fluorescence images by flattening z-stacks, denoising the images and removing dim and dead cells. The end result is a *.cells file that contains the image data on a cell-by-cell basis.

Input Data Parameters

1px in nm

How many nanometers correspond to 1px. This is useful for your dataset to have the proper scale and allow downstream processing steps to access this information. If blank no conversion will be used and all downstream values will be in px.

Stacking

Channel to use

For multichannel images (like Tiff or DeltaVision files), this selects the channel to use; this can be ignored if the image has only one channel. A channel will usually contain data on a single reporter (e.g. mCherry channel, GFP channel etc.) The channels are 0 based, that means the first channel is 0, the second is 1 and so on.

Stacking Type

Assuming that the images contain z stacks in each channel, the first step is to project this stack to a single image.
"Use Max Value": Typically for every pixel position the brightest value from all z planes is taken, creating a max image.

"Use Mean Value": Alternatively we can average the pixel value across all z-planes in a given position.

It is recommended to use the max setting. Because foci usually are located in only a single plane, using the mean setting will "dilute them out". Also depending on setting of the z-planes, images at the top and bottom of cells may often be very often dim, since they may be out of focus/partially outside the cell, which again dilutes out the features.

Z Planes to use

Governs which z-planes to use. Leave empty to use all. Otherwise you can define ranges with a colon and commas. Note that indices start from 0.

Example:
0:3 selects the z planes: 0,1,2,3
3:4,7:8 selects the z planes: 3,4,7,8
1,2,3 selects the z planes: 1,2,3

Denoise

Denoising Strength

Large values perfectly remove noise, but also may remove image details. Smaller values preserve details, but also will preserve some noise. If set to 0, no denoising occurs. The denoising used here is the non-local means denoising [2]. Keep in mind that images are reduced to 8 bit, for performance reasons. This is usually the only parameter that needs to be set, and you can leave the other two parameters as they are.

Template Window Size

Size in pixels of the template patch that is used to compute weights. Should be odd. Recommended value 7 pixels.

Search Window Size

Size in pixels of the window that is used to compute weighted average for given pixel. Should be odd. Affects performance linearly: greater searchWindowsSize = greater denoising time. Recommended value 21 pixels.

Cell Selection

Border

Every cell closer than this value in pixels to the image border will be automatically discarded. This is useful as cells at the very edge of the image border are usually not completely visible. Some microscopes also produce artifacts around the borders due to the use of deconvolution.

Intensity Range (Avg in Cell)

Dim cells suffer disproportionately from noise and make detection of foci imprecise. Very bright cells might be oversaturated and equally impair analysis. Those cells can be discarded by adjusting this intensity range. The brightness values are normalized 0 - 1. In this case the <u>mean</u> intensity has to lie within the bounds of this parameter.

Intensity Range (Max in Cell)

Dim cells suffer disproportionately from noise and make detection of foci imprecise. Very bright cells might be oversaturated and equally impair analysis. Those cells can be discarded by adjusting this intensity range. The brightness values are normalized 0 - 1. In this case the <u>maximum</u> intensity must lie within the bounds of this parameter.

Mask Shift

Due to optical effects or alignment problems of a given microscope, the fluorescence channel might theoretically be shifted compared to the brightfield channel. This can be a problem if features of interest are located close to the cell wall/membrane. You can shift the whole mask by a few pixels to avoid this problem.

You can address this problem (if present) by using the following technique. First set the contrast on the top to a value where the fluorescence channel is very clearly visible and a little too bright (set contrast slider to 0-0.5 for example). Then hold the “2” key to temporarily make the cell outlines invisible. Lastly, slide your Ref opacity back and forth to make the cell outline image appear and disappear. You will get a feel about how close your fluorescence channel features are to the cell outlines and you might notice that some are very close to the cell boundaries, or even slightly outside of these. In this case, you can use the mask shift to resolve the problem. You should however investigate why your data contains this problem in the first place, since it should not.

Input consists of two numbers for X and Y shift respectively, separated by a semicolon. e.g., "5;-5". Leave empty if you do not need to use shifting for your data/microscope.

Positive numbers shift to bottom and right. Units are pixels. The output of this pipeline will store the shifted versions of cells.

Mask Tightening

Shrinking allows the mask to be tightened to the background of the fluorescence signal. This can be beneficial, if your cells clump together or if the boundary was not shrunk initially. In most cases you can skip this step.Shrinking uses the active contours model [3]; please refer to this paper for precise explanation on parameters. The contours are often referred to as “snake”.

Alpha

Snake length shape parameter. Higher values make the snake contract faster.

Smoothness (a.k.a. Beta)

Beta or Snake smoothness shape parameter. Higher values make the snake smoother.

Gamma

Explicit time stepping parameter.

Iterations

Maximum iterations to optimize snake shape.

Model Training

Trains the model to recognize foci of desired size. The result is a *.model file containing the trained weights for the foci recognition as well as size limits of foci (see “Foci Circumference in px” Parameter). This model file will only work with data of the same resolution and should only be used for foci of the same type (e.g. only stress granules).

Foci Candidates

Foci Circumference in px

Min/Max circumference of the foci in px. This is an approximate value that needs to be set according to the size of features in question. Choose the max value that is small enough not to encompass unnecessarily big areas or multiple foci. Choose the min value to be as big as possible but small enough to capture the smallest foci.

The actual sizes of the candidate foci will lie in the range between these two values and are determined automatically.

To set this parameter optimally, look at different cells by selecting them in the main view and observing whether or not the foci you are trying to detect are correctly recognized and only rarely merge. While you can later resolve some of the merging, it is best to set this parameter rather tighter to prevent the merging from happening.

Granularity

It is not recommended to change this parameter, since it has only a very minor effect. It is kept mostly for performance reasons on large datasets, since lower granularity makes the algorithm faster, but less precise.

Contour Loops are analyzed by first finding Granularity contours at different levels of intensity. Out of these, closed loops are picked out as candidates.

The algorithm governs how close the shortest and longest contours will be to the desired circumference. The higher the granularity is, the more the contours found will match the desired circumference, but also the contour extraction will be (~linearly) slower. Consider lowering this parameter if your dataset is very large and contour detection takes a long time, then identify the best parameters, and before exporting set it back to a higher value.

Labeling

Random Order

Determines whether or not the cells for labeling are presented in a random order. If labeling the same dataset more than once, it makes sense to randomize it. Later you could compare your own labeling to itself to assess variability in your quantification.

Show Training Curve

Shows the training curve on a successively increasing subset of labeled data. This allows you to assess whether or not it is worth adding more labels to the training set. Given a very small subset of data the model’s performance will be poor. Generally, as the set size increases so does the performance. At some point the curve levels off and adding more data does not lead to a significant increase in performance. You can stop adding data if you see the curve leveling off. Generating this curve might take some time and is therefore optional.

Foci Detection (via model)

Uses the model from a previous pipeline and applies it to the detection of foci. Additional foci can be added (or removed) manually as part of this pipeline. Trains the model to recognize foci of desired size.

Input Data Parameters

1px in nm

How many nanometers correspond to 1px. This is useful for your dataset to have the proper scale and allow downstream processing steps to access this information. If blank no conversion will be used and all downstream values will be in px.

Use subset in %

If your dataset is large, you might only want to process the first x% of it, to get a feel for the result. At this point you might determine to use another model, pipeline, or process the whole dataset. Use this slider to adjust the percentage of cells to process.

Adjust Foci Size

If you feel the determined foci sizes are too big or too small, you can adjust all of them by this factor. Keep in mind that if you make the adjustment bigger, it will lead to merging of adjacent foci into a single one.

Cell Outlines

Simply toggles whether the UI displays the cell outlines.

Foci Detection (via Parameters)

Uses a set of intuitive parameters to exclude and include foci. Additionally, a manual processing of the foci can be done. This pipeline is useful when your cells have few and very clear foci (i.e. high signal to noise, and/or when reliably identifying foci above a certain threshold signal is feasible). It is a lot faster than training a model, but requires a little bit of manual work.

Input Data Parameters

1px in nm

How many nanometers correspond to 1px. This is useful for your dataset to have the proper scale and allow downstream processing steps to access this information. If blank no conversion will be used and all downstream values will be in px.

Use subset in %

If your dataset is large, you might only want to process the first x% of it, to get a feel for the result. At this point you might determine to use another model, pipeline, or process the whole dataset. Use this slider to adjust the percentage of cells to process.

Foci Candidates

Foci Circumference in px

Min/Max circumference of the foci in px. This is an approximate value that needs to be set according to the size of features in question. Choose the max value that is small enough not to encompass unnecessarily big areas or multiple foci. Choose the min value to be as big as possible but small enough to capture the smallest foci. The actual sizes of the foci will lie in the range between these two values and are determined automatically.

To set this parameter, look at different cells by selecting them in the main view and observing whether or not the foci you are trying to detect are correctly recognized and only rarely merge. While you can later resolve some of the merging, it is best to set this parameter rather tightly to prevent the merging from happening.

Granularity

It is not recommended to change this parameter, since it has only a very minor effect; it is kept mostly for performance reasons on large datasets. Lower granularity makes the algorithm faster, but less precise.

Contour Loops are analyzed by first finding Granularity contours at different levels of intensity. Out of these closed loops are picked out as candidates.

It governs how close the shortest and longest contours will be to the desired circumference. The higher the granularity is, the more the contours found will match the desired circumference, but also the contour extraction will be (~linearly) slower. Consider lowering this parameter if your dataset is very large and contour detection takes a long time, then identify the best parameters, and before exporting set it back to a higher value.

Foci Detection Params

Adjust Foci Size

If you feel the determined foci sizes are too big or too small, you can adjust all of them by this factor. Keep in mind that if you make the adjustment bigger, it will lead to merging of adjacent foci into a single one.

Normalized Brightness (NB)

Filters by normalized brightness (i.e., brightness inside the cell is normalized 0 - black, to 1 - white). For all foci the average normalized brightness inside its area needs to lie inside these bounds.

Setting this setting to for example 0.5 - 1 will remove all foci that are dimmer than a gray (50% black). This setting is useful if you have many foci and need to reduce the amount of foci, by focussing only on the brightest inside the cell.

However since the brightness is normalized the absolute signal from the microscope might be close to 0 but appear a “bright” gray. To exclude by absolute or raw brightness use the next parameter.

Raw Brightness (RB)

For all foci the average absolute (i.e., unscaled, coming from microscope) brightness inside its area needs to lie inside these bounds.

Use this setting to exclude cells with low absolute signal. Usually at this point you will have excluded cells with low or close to no signals, but if not there is an opportunity here to exclude anything with a low original brightness, since these foci are often results of simply artifacts or noise.

Min Brightness Drop

The brightness ratio between the brightest point of the focus and its outline. A value of for example 4 signifies that only foci are considered where the peak brightness is at least 4 times brighter than the outline of the focus.

Keep in mind that this parameter is of course entangled with the size of the foci that can be influenced by the “adjust foci size”, so the bigger your foci are, the larger the drop between center and outline will naturally be.

Colocalization

Performs different colocalization analyses on two labeled datasets.

Input Data Parameters

Name Channel 1, Name Channel 2

You can give a name to Dataset 1 or 2, e.g. "Stress Granules" pr “P-Bodies”. This will make the subsequent evaluation easier to understand and is highly recommended.

1px in nm

How many nanometers correspond to 1px. This is useful for your dataset to have the proper scale and allow downstream processing steps to access this information. If blank no conversion will be used and all downstream values will be in px.

Dataset Alignment

- no parameters at present -

Cell Overview

Color Combination

This is an aesthetic setting only. Pick a color combination of the two channels to use in your images.

Channel Shift

Some microscopes might produce slight shifts in images depending on wavelength and other microscope parameters; this can affect colocalization of especially small features significantly. This setting will shift the second channel by the given amount.

Input consists of two numbers for X and Y shift respectively, separated by semicolon. e.g. "200;-200". Leave empty or set to 0 if you do not need to use shifting for your data/microscope.

Units are either nanometers or pixels, depending on whether the scale was provided in the Data Input step to convert pixels to nanometers. Positive numbers shift to bottom and right.

Normalize Brightness

Signal strength varies by cell. While some cells might have the full spectrum of a signal from 0 to 1, others might only use 10% of it (0 - 0.1 for example). Normalizing will artificially normalize the signal to unit range: 0-1. This makes all foci clearly visible regardless of signal strength. However, cells with no signal/no foci will appear fully bright after normalization, as if the signal strength is close to 1 over the whole cell.

The pearson correlation is invariant to normalization. Thus this setting is a purely visual setting, to more clearly see shapes/locations of foci.

Graphs

Fit (for scatter plot only)

If a regression function can be fit to the data, you can choose between different models for it. A fit is not always possible, in that case you will not see a curve and a NaN is displayed as R^2 value.
Linear:y = a*x + b
Exponential:y = a * exp(bx)
Polynomial: y = a*x^p + b*x^(p-1) ... with p being the order of the polynomial
Logarithmic:y = a + b*ln(x)
Power:y = a * x^b

Bins (for all bar graphs)

The number of bins to display

References

[1]
Frangi, A. F., Niessen, W. J., Vincken, K. L. & Viergever, M. A. Multiscale vessel enhancement filtering. *Lect. Notes Comput. Sci. (including Subser. Lect. Notes Artif. Intell. Lect. Notes Bioinformatics)* **1496**, 130–137 (1998).

[2]
Buades, A., Coll, B. & Morel, J.-M. Non-Local Means Denoising. *Image Process. Line* **1**, 208–212 (2011).

[3]
Kass, M., Witkin, A. & Terzopoulos, D. Snakes: Active contour models. *Int. J. Comput. Vis. 1987 14* **1**, 321–331 (1988).

Features*	HARLEY	Fiji/ImageJ	CellProfiller
Batch processing	Yes	No	Yes
Trainable model	Yes	No	Yes (via CellProfiler Analyst, though high learning curve)
Detailed statistical evaluation of foci co-localization	Yes	Limited	Limited
Concise User Interface with explanations	Yes	No	No
Manual intervention opportunities to fix pipeline errors	Yes	No	Limited
Contour-based foci detection	Yes	No	No
Configuration requirement	None, use as is	Mostly finding Plugins	Extensive (building or finding pipelines)
Open Source	Yes	Yes	Yes
Pipeline based model allowing storage of intermediary results	Yes	No	Limited to type of results
Tailored to yeast images	Yes	No	No

Supplementary Table S1: Features of HARLEY in comparison to other common microscopy analysis tools.

*Some features, in some applications, will not always be readily classifiable into clear yes/limited/no groups. Generally, this table reflects current features of HARLEY, Fiji and CellProfiller to the best of our knowledge
